# Simple parameters predicting extrahepatic recurrence after curative hepatectomy for hepatocellular carcinoma

**DOI:** 10.1038/s41598-021-92503-6

**Published:** 2021-06-21

**Authors:** Jae Hyun Yoon, Won Jae Lee, Sun Min Kim, Kwang Tack Kim, Sung Bum Cho, Hee Joon Kim, Yang Seok Ko, Hyun Yi Kook, Chung Hwan Jun, Sung Kyu Choi, Ban Seok Kim, Seo Yeon Cho, Hye-Su You, Yohan Lee, Seyeong Son

**Affiliations:** 1 Department of Gastroenterology, Chonnam National University Hospital and Medical School, Gwangju, 61469 South Korea; 2 Department of Gastroenterology, Hwasun Chonnam National University Hospital and Medical School, Hwasun, South Korea; 3 Department of Surgery, Chonnam National University Hospital and Medical School, Gwangju, South Korea; 4 Department of Surgery, Hwasun Chonnam National University Hospital and Medical School, Hwasun, South Korea; 5 Department of Nursing, Chonnam National University, Gwangju, South Korea; 6 Department of Internal Medicine, Mokpo Hankook Hospital, Mokpo, 58643 South Korea

**Keywords:** Cancer, Gastroenterology, Risk factors

## Abstract

Extrahepatic recurrence (EHR) after curative hepatectomy for hepatocellular carcinoma (HCC) is associated with a poor prognosis. We investigated the features of EHR and identified its predictive factors. This retrospective study included 398 treatment-naive patients who underwent curative hepatectomy for HCC at two tertiary hospitals. Multivariate Cox-regression analysis was performed to identify the variables associated with EHR. EHR was diagnosed in 94 patients (23.6%) over a median follow-up period of 5.92 years, most commonly in the lungs (42.6%). The 5-/10-year cumulative rates of HCC recurrence and EHR were 63.0%/75.6% and 18.1%/35.0%, respectively. The median time to EHR was 2.06 years. Intrahepatic HCC recurrence was not observed in 38.3% of patients on EHR diagnosis. On multivariate analysis, pathologic modified Union for International Cancer Control stage (III, IVa), surgical margin involvement, tumor necrosis, sum of tumor size > 7 cm, and macrovascular invasion were predictive factors of EHR. Four risk levels and their respective EHR rates were defined as follows: very low risk, 1-/5-year, 3.1%/11.6%; low risk, 1-/5-year, 12.0%/27.7%; intermediate risk, 1-/5-year, 36.3%/60.9%; and high risk, 1-year, 100.0%. Our predictive model clarifies the clinical course of EHR and could improve the follow-up strategy to improve outcomes.

## Introduction

Despite recent advances in the diagnosis and treatment of hepatocellular carcinoma (HCC), HCC continues to be associated with poor prognosis, presenting the third highest cancer-related mortality rate worldwide^1,2^. Curative hepatectomy remains the treatment choice for such cases, especially in settings where liver transplantation is not feasible^3^. However, the long-term prognosis after curative hepatectomy remains unsatisfactory, with the 5-year rate of HCC recurrence ranging between 60 and 70%^4,5^. Therefore, identifying risk factors of HCC recurrence and standardizing the perioperative management protocol could be important to improve long-term prognosis after curative hepatectomy for HCC.

According to the current practice, curative hepatectomy is indicated over other local therapies, such as radiofrequency ablation (RFA), for patients with advanced HCC who have larger size tumors and/or presence of microvascular tumor invasion. The more advanced disease status of patients who undergo curative hepatectomy could explain the comparatively higher risk of HCC recurrence after curative hepatectomy than RFA. Current treatment guidelines recommend surveillance after treatment, with curative hepatectomy or RFA, including abdominal computed tomography (CT) and measurement of serum alpha fetoprotein (AFP) levels^6,7^. However, this recommendation does not consider the differences in the risk of recurrence between patients treated using curative hepatectomy and those treated with RFA^8^. Moreover, although intrahepatic recurrence (IHR) is the most common type of recurrence, extra-hepatic recurrence (EHR) is possible, with the most common sites of EHR being the lungs, lymph nodes, and bones, which could be difficulty evaluated using conventional abdominal CT imaging^9–11^. Considering the aggressive nature of metastatic hepatic tumors and the limited treatment options for recurrent HCC, the prognosis for patients with EHR is generally worse than that for those with IHR. Despite the dismal prognosis of EHR, few studies have showed improved outcomes with mestastasectomy in selected patients^12,13^. Thus, early identification would be important to improve the oncological outcomes and survival. However, at present, there are insufficient data on the clinical course and pathological progression after curative hepatectomy for HCC to identify the predictive factors of EHR. Accordingly, we aimed to determine the risk factors of EHR among patients who had undergone curative hepatectomy as the initial treatment for HCC and to use these risk factors to construct a simple parametric model to predict EHR.

## Results

### Baseline characteristics of enrolled patients

**Figure 1:**
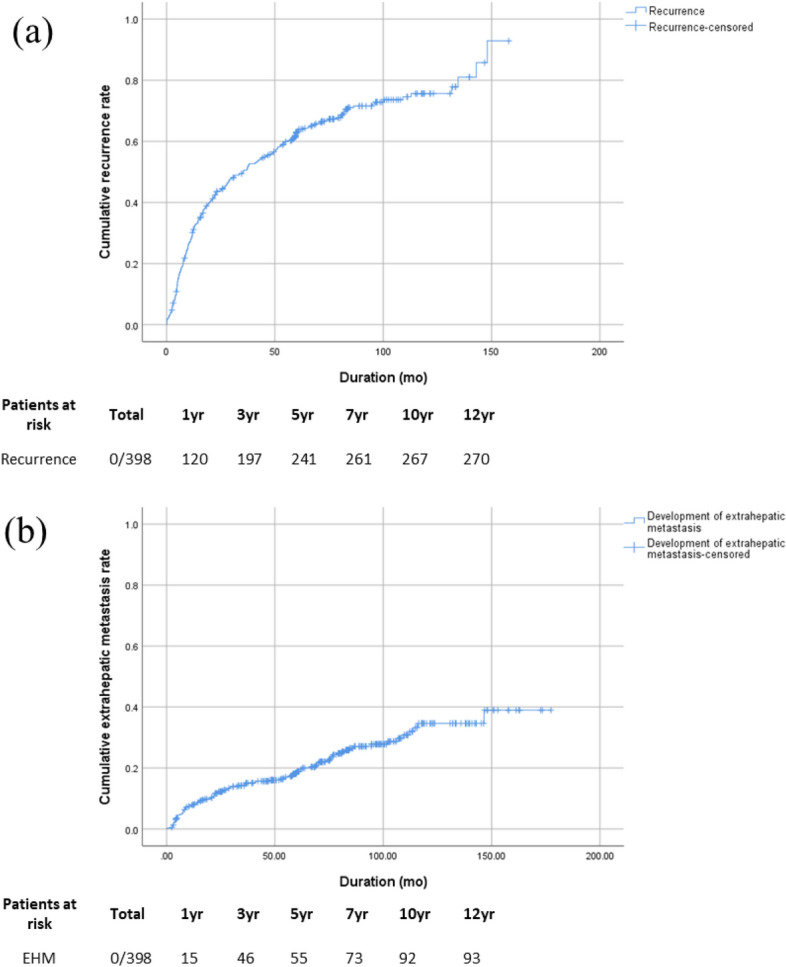
Recurrence curves of hepatocellular carcinoma (HCC) after surgical resection (**a**): Cumulative rates of HCC recurrence; the 1-, 3-, 5-, 7-, and 10-year cumulative rates of recurrence were 30.4%, 50.5%, 63.0%, 71.0%, and 75.6%, respectively; (**b**): Cumulative rates of extrahepatic recurrence (EHR); the 1-, 3-, 5-, 7-, and 10-year cumulative rates of EHR were 7.9%, 14.2%, 18.1%, 25.5%, and 35.0%, respectively.

EHR was identified in 94 (23.6%) out of 398 enrolled patients. The 10-year cumulative rate of HCC recurrence was 75.6%, with a rate of 35.0% for EHR (Fig. 1). Compared to those without EHR, those with EHR were younger and had a higher serum alkaline phosphatase level, a lower serum albumin level, absence of fatty change in the liver, and a more advanced HCC stage (Table 1). The serum AFP level at the time of first recurrence of HCC after curative hepatectomy was higher and the time interval to the first recurrence was also significantly shorter in the EHR group.

**Table 1:** Baseline characteristics of the enrolled patients.

		Patients without extrahepatic recurrence (n = 304)	Patients with extrahepatic recurrence (n = 94)	*p*-value
Age (years)	59.11 ± 10.02		56.01 ± 10.45	**0.010**
Male (n, %)	259 (85.2)		85 (90.4)	0.196
**Etiology of liver cirrhosis, n (%)**				0.897
HBV*/HCV Alcohol/combined NASH/unknown	176 (63.1)/21 (7.5) 24 (8.6)/17 (6.1) 1 (0.4)/40(14.4)		56 (65.9)/6 (7.1) 10 (11.8)/4 (4.7) 0 (0.0)/9 (10.6)	
ALP (U/L)	86.88 ± 28.21		99.25 ± 49.72	**0.023**
Albumin (mg/dL)	4.35 ± 0.48		4.13 ± 0.45	** < 0.001**
ALBI grade ≥ 2, n (%)	47 (15.5)		23 (24.7)	0.041
ICG R15	10.33 ± 7.62		10.99 ± 8.08	0.496
Preoperative serum AFP (IU/mL)				0.363
≤ 400 > 400	235 (80.5%) 57 (19.5%)		70 (76.1%) 22 (23.9%)	
Tumor size	4.18 ± 2.41		5.16 ± 3.69	** < 0.001**
Tumor numbers	1.26 ± 0.83		1.32 ± 0.79	0.525
**BCLC stage, n (%)**				**0.045**
0/A / ≥ B	34 (11.2)/234 (77.2)/35 (11.6)		6 (6.4)/68 (72.3)/20 (21.3)	
**Pathological mUICC stage, n (%)**				**0.012**
I/II / ≥ III	43 (14.5)/183 (61.6)/71 (23.9)		7 (8.0)/49 (56.3)/31 (35.6)	
**Radiological mUICC stage, n (%)**				**0.001**
I/II / ≥ III	48 (15.8)/205 (67.7)/50 (16.5)		10 (10.6)/59 (62.8)/212 (26.6)	
Beyond Milan criteria, n (%)	75 (24.8)		45 (47.9)	** < 0.001**
Metastatic lymph nodes, n (%)	0 (0.0)		2 (2.1)	**0.011**
Macrovascular invasion, n (%)	6 (2.0)		8 (8.5)	**0.003**
**mUICC T stage at 1st recurrence, n (%)**				< 0.001
0/1 2 /3 4	0 (0.0)/81 (45.8) 67 (37.9)/25 (14.1) 4 (2.3)		6 (6.5)/19 (20.7) 32 (34.8)/22 (23.9) 13 (14.1)	
Serum AFP at 1st recurrence	209.12 ± 1 098.83		1,225.05 ± 5 775.70	**0.045**
Hospital stay, days (median, range)	13 (5–69)		13 (4–60)	**0.015**
Time to first recurrence, months (median, range)	42.54 (0.16–157.91)		10.14 (0.23–100.34)	** < 0.001**
Follow-up duration, months (median, range)	75.21 (2–177)		38.93 (3–150)	**0.857**

Values are presented as mean ± SD.

*SD* standard deviation, *HBV* hepatitis B virus, *HCV* hepatitis C virus, *AST* aspartate transaminase, *ALT* alanine transaminase, *ALP* alkaline phosphatase, *AFP* alpha-fetoprotein, *BCLC* Barcelona Clinic Liver Cancer, *mUICC* modified Union for International Cancer Control.

*Patients with suppressed HBV DNA (HBV DNA < 200 IU/mL)^42^ at pre-operative state had lower rates of EHR (11.1% vs. 28.8%, p = 0.047).

### Clinical features of patients with EHR

The most common site of the first HCC recurrence in the EHR group was intrahepatic (66.0%), with the most common initial site of EHR being the lungs (42.6%), followed by the lymph nodes (19.1%), peritoneum (18.1%), and bones (14.9%) (Supplementary Table S1). In half of the cases, EHR was confined to the abdominal cavity, identified by abdominal imaging, while in the other 48.9% of cases, EHRs were identified within the thoracic cavity, including the lungs and the bony structures of the thoracic spine. The median time to EHR was 2.06 years. At the time of EHR diagnosis, 36 patients (38.3%) had no IHR. At the time of first HCC recurrence, EHR was identified in 32 patients (34.0%).

### Comparison of surgical findings between patients with and without EHR

Microvascular and serosal invasion were more prevalent among patients with EHR than in those without (16.9 *versus* 29.8% and 2.1 *versus* 6.5%, respectively) (Table 2). Moreover, the presence of satellite nodules and tumor necrosis in resected specimens was more prominent in patients with EHR than in those without (13.0 *versus* 31.2% and 46.9 *versus* 68.8%, respectively).

**Table 2:** Comparison of surgical findings in patient groups with and without extrahepatic recurrence.

	Patients without extrahepatic recurrence (n = 304)	Patients with extrahepatic recurrence (n = 94)	*p*-value
Margin involvement, n (%)	9 (3.0)	6 (6.5)	0.134
Microvascular invasion, n (%)	51 (16.9)	28 (29.8)	**0.006**
Serosal invasion, n (%)	6 (2.1)	6 (6.5)	**0.034**
Bile duct invasion, n (%)	1 (0.3)	2 (2.25)	0.085
Capsule formation, n (%)	211 (73.3)	64 (68.8)	0.405
Multicentricity, n (%)	34 (11.7)	9 (9.7)	0.593
Satellite nodule, n (%)	38 (13.0)	29 (31.2)	** < 0.001**
Underlying liver cirrhosis, n (%)	201 (66.1)	61 (64.9)	0.827
Intrahepatic metastasis, n (%)	3 (1.0)	2 (2.1)	0.830
Necrosis, n (%)	136 (46.9)	64 (68.8)	** < 0.001**
Haemorrhage, n (%)	139 (47.9)	53 (57.0)	0.121
Fatty change, n (%)	109 (38.0)	29 (31.9)	0.291
**Cell type, n (%)**			0.213
Clear type Hepatic type Classic type	53 (17.5) 271 (89.4) 240 (79.2)	22 (23.4) 89 (94.6) 70 (74.5)	
**Major Edmondson Steiner grade, n (%)**			0.336
1/2 3/4	14 (4.8)/152 (52.2) 116 (39.9)/9 (3.1)	4 (4.3)/39 (41.9) 46 (49.5)/4 (4.3)	
**Worst Edmondson Steiner grade, n (%)**			0.665
1/2 3/4	2 (0.7)/46 (15.8) 178 (61.2)/65 (22.3)	2 (2.2)/14 (15.1) 55 (59.1)/22 (23.7)	

### Comparison of characteristics between patients with early and non-early EHR

**Figure 2:**
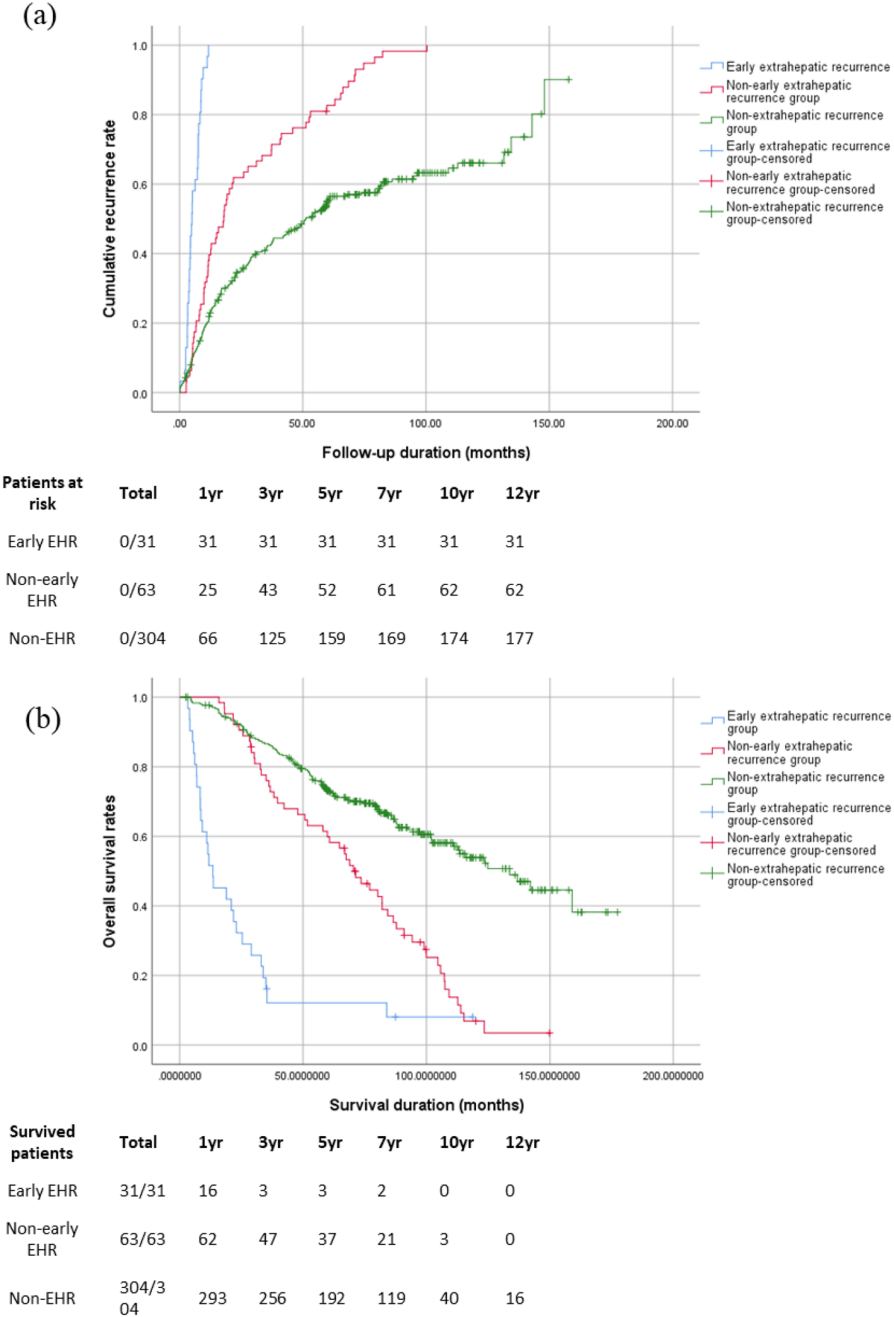
Comparison of the (**a**) intra- and/or extra-hepatic recurrence rate of hepatocellular carcinoma (HCC) and (**b**) overall survival rates between the early extrahepatic recurrence (EHR), non-early EHR, and non-EHR groups. The 6-month cumulative recurrence rates among the early EHR, non-early EHR, and non-EHR groups were 77.7%, 39.7%, and 21.9%, respectively. The 1-year, 5-year, and 10-year overall survival rates were 51.6%, 9.7%, and 0% in the early EHR group; 98.4%, 58.7%, and 4.8% in the non-early EHR group; and 96.4%, 63.2%, and 13.2% in the non-EHR group, respectively (*p* < 0.001).

Both the radiologic and pathologic mUICC stages were more advanced in the early EHR than in the non-early EHR group (Supplementary Table S2). The early EHR group also had a markedly shorter recurrence-free-survival (RFS) and survival rates compared to the non-early EHR and non-EHR groups (Fig. 2). Moreover, the proportion of tumors with a mUICC stage ≥ III at the time of first recurrence was larger in the early EHR than in the non-early EHR group. Especially, in 54.8% of patients in the early EHR group, EHR was the first presenting recurrence after curative hepatectomy.

### Analysis of factors associated with EHR

In the multivariate analysis, the following factors were retained as independent predictors of EHR: pathologic mUICC stage (III, IVa) (Hazard ratio [HR]: 2.664, *p* = 0.013), surgical margin involvement (HR: 3.040, *p* = 0.040); tumor necrosis on pathological assessment of resected specimens (HR: 1.797, *p* = 0.037); sum of tumor size > 7 cm (HR: 2.481, *p* = 0.014); macrovascular invasion on imaging studies (HR: 3.295, *p* = 0.011 (Table 3). Regarding the factors associated with early EHR, serum alkaline phosphatase (ALP) > 120 U/L, a pathological mUICC stage III or IVa, surgical margin involvement, absence of fatty change in the liver, and macrovascular invasion were found to be closely associated on multivariate analysis (Table 4).

**Table 3:** Univariate and multivariate analyses of factors associated with extrahepatic metastasis.

	Univariate analysis		Multivariate analysis	
	HR (95% CI)	*p*-value	HR (95% CI)	*p*-value
Serum alkaline phosphatase > 130 U/L	2.800 (1.222–6.416)	0.015		
ALBI grade ≥ 2	1.889 (1.175–3.036)	0.009		
Pathologic mUICC stage (III, IVa)	2.398 (1.586–3.626)	< 0.001	**2.664 (1.232–5.763)**	**0.013**
Multiple tumors^a^	1.980 (1.183–3.313)	0.009		
Major Edmondson Steiner grade ≥ 3	1.529 (1.016–2.299)	0.042		
Surgical margin involvement	2.749 (1.200–6.298)	0.017	**3.040 (1.050–8.804)**	**0.040**
Venous/lymphatic involvement	2.043 (1.312–3.180)	0.002		
Serosa invasion	3.167 (1.380–7.271)	0.007		
Bile duct invasion	5.720 (1.401–23.350)	0.015		
Satellite nodule	2.632 (1.694–4.090)	< 0.001		
Tumor necrosis	2.333 (1.504–3.620)	< 0.001	**1.797 (1.035–3.118)**	**0.037**
Sum of tumor size > 7 cm	3.792 (2.394–6.004)	< 0.001	**2.481 (1.198–5.135)**	**0.014**
Beyond Milan criteria	2.832 (1.881–4.263)	< 0.001		
Macrovascular invasion	4.005 (1.934–8.295)	< 0.001	**3.295 (1.310–8.292)**	**0.011**
BCLC stage C	2.271 (1.382–3.731)	0.001		
Serum AFP ≥ 50,000 IU/mL	5.519 (1.347–22.621)	0.018		

*HR* hazards ratio, *CI* confidence interval, *mUICC* modified Union for International Cancer Control, *CT* computed tomography, *MRI* magnetic resonance imaging, *BCLC* Barcelona Clinic Liver Cancer, *AFP* alpha-fetoprotein.

^a^Number of tumors examined at pathologic findings.

**Table 4:** Univariate and multivariate analyses of factors associated with early extrahepatic recurrence.

	Univariate analysis		Multivariate analysis	
	HR (95% CI)	*p*-value	HR (95% CI)	*p*-value
Serum ALP > 120 U/L	2.790 (1.279–6.084)	0.010	**2.362 (1.018–5.478)**	**0.045**
Pathologic mUICC stage (III, IVa)	3.418 (1.634–7.151)	0.001	**2.610 (1.154–5.901)**	**0.021**
Worst Edmonson Steiner grade ≥ 4	2.221 (1.063–4.643)	0.034		
Surgical margin involvement	2.991 (1.043–8.576)	0.041	**4.035 (1.280–12.725)**	**0.017**
Venous/lymphatic involvement	1.890 (0.925–3.860)	0.081		
Absence of fatty change	3.582 (1.249–10.276)	0.018	**3.246 (1.114–9.461)**	**0.031**
Sum of tumor size > 7 cm	2.142 (1.055–4.347)	0.035		
Macrovascular invasion	3.818 (1.559–9.351)	0.003	**3.207 (1.169–8.799)**	**0.024**

*HR* hazards ratio, *CI* confidence interval, *ALP* alkaline phosphatase, *mUICC* modified Union for International Cancer Control, *AFP* alpha-fetoprotein.

### Prediction of EHR

**Figure 3:**
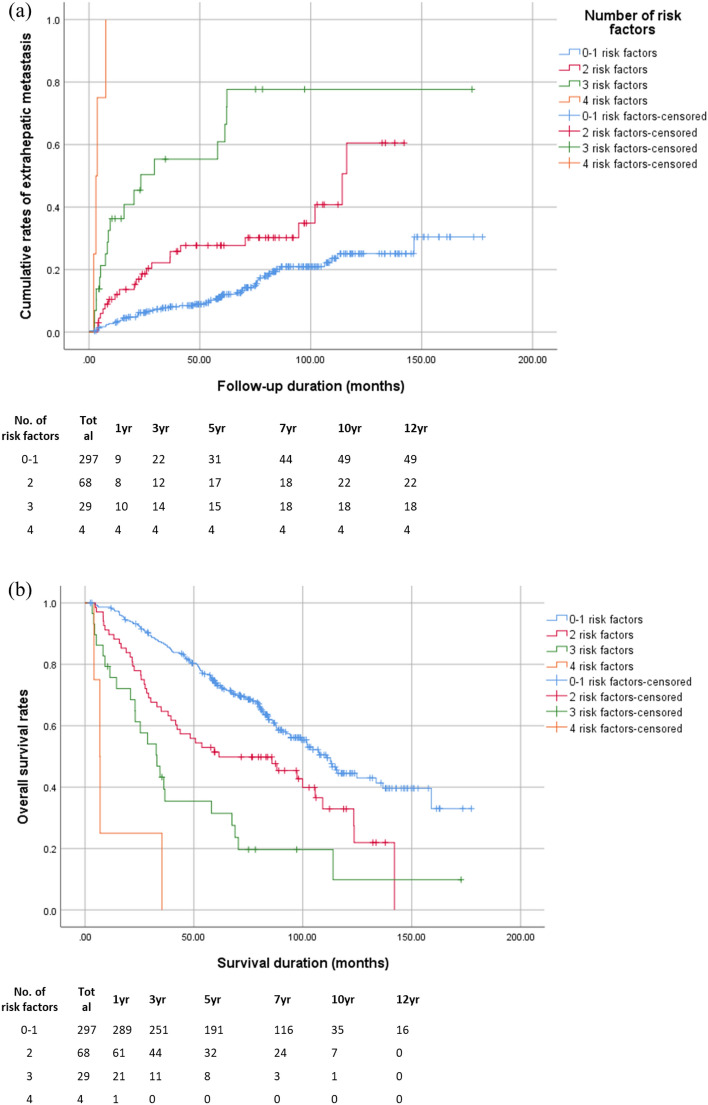
(**a**) Cumulative rate of extrahepatic recurrence and (**b**) overall survival rates after curative hepatectomy, stratified by the number of risk factors present.

Based on our multivariate analyses, the following five variables were used to build a parametric model to predict EHR. Then, the risk of EHR was stratified into four levels based on the number of predictive factors present, as follows: very low risk, 0–1 risk factors; low risk, 2 risk factors; intermediate, 3 risk factors; and high, ≥ 4 risk factors. Then, cumulative rate of EHR was calculated using the Kaplan–Meier survival curve analysis for each risk level (Fig. 3a). The 1-, 3-, 5-, and 10- year cumulative rates of EHR were significantly related to the numbers of risk factors present: very low risk: 3.1%, 7.3%, 11.6%, and 25.1%, respectively; low risk: 12.0%, 22.2%, 27.7%, and 60.5%, respectively; intermediate risk: 36.3%, 55.3%, 60.9%, and 73.7%, respectively; and high risk: 100.0% at 1st year. Furthermore, the overall survival rates showed a significant correlation with the risk level of EHR (Fig. 3b). The 1-, 3-, 5-, and 10- year overall survival rates were as follows: very low risk: 97.3%, 84.5%, 64.3%, and 11.8%, respectively; low risk: 89.7%, 64.7%, 47.1%, and 10.3%, respectively; intermediate risk: 72.4%, 37.9%, 27.6%, and 3.4%, respectively; and high risk: 25.0% at 1st year and no survived patient from 35 months. We assessed the discriminative ability of the model with Harrell’s C index, which showed a value of 0.685 (95% confidence interval [CI], 0.624–0.745)^14,15^. The median times to EHR development were 71.41, 50.01, 14.47, and 3.44 months for the very-low, low, intermediate, and high-risk levels, respectively.

### Analysis of factors associated with the overall survival rates

To analyze the association with EHR with the overall survival rate, we conducted Cox-regression analysis including many potential risk factors, such as EHR (Table 5). On multivariate analysis, pathologic modified Union for International Cancer Control (mUICC) stages III and IVa (HR: 3.118, *p* < 0.001), surgical margin involvement (HR: 4.847, *p* < 0.001), initial tumor stage beyond Milan criteria (HR: 2.242, *p* = 0.012), and presence of EHR (HR: 4.723, *p* < 0.001) were found to be associated with the overall survival rates.

**Table 5:** Univariate and multivariate analyses of factors associated with overall survival.

	Univariate analysis		Multivariate analysis	
	HR (95% CI)	*p*-value	HR (95% CI)	*p*-value
Serum albumin < 3.7 mg/dL	3.012 (1.674–5.419)	< 0.001		
ALBI grade ≥ 2	2.216 (1.328–3.699)	0.002		
Pathologic mUICC stage (III, IVa)	3.167 (1.980–5.066)	< 0.001	**3.118 (1.682–5.782)**	** < 0.001**
Multiple tumors^a^	4.004 (2.181–7.352)	< 0.001		
Surgical margin involvement	6.013 (2.980–12.135)	< 0.001	**4.847 (1.875–12.532)**	**0.001**
Venous/lymphatic involvement	2.442 (1.490–4.002)	< 0.001		
Serosa invasion	5.083 (2.321–11.132)	< 0.001		
Bile duct invasion	7.365 (1.799–30.152)	0.005		
Multicentricity	2.391 (1.305–1.380)	0.005		
Satellite nodule	2.243 (1.329–3.786)	0.002		
Tumor necrosis	2.173 (1.307–3.612)	0.003		
Sum of tumor size > 7 cm	3.224 (1.881–5.526)	< 0.001		
Beyond Milan criteria	2.486 (1.548–3.991)	< 0.001	**2.242 (1.191–4.219)**	**0.012**
Presence of EHR	3.994 (2.495–6.395)	< 0.001	**4.723 (2.512–8.882)**	** < 0.001**

*HR* hazards ratio, *CI* confidence interval, *mUICC* modified Union for International Cancer Control, *EHR* extrahepatic recurrence.

^a^Number of tumors examined at pathologic findings.

## Discussion

Based on the data of many patients who underwent curative hepatectomy for HCC, we proposed a simple parametric model predicting the risk of EHR development. This model is straightforward and easy-to-use, and consists of five easy-to-obtain variables that constitute the essentials of pre- and post-operative clinical parameters and the postoperative pathologic findings. Because of the lack of current consensus on follow-up strategies for the detection of EHR after resection, our prediction models may aid in monitoring patients for individual risk and in appropriately assigning patients for participation in clinical trials for postoperative adjuvant therapy (e.g., patients would be categorized as intermediate or high risk, if they present a predicted 5-year EHR rate of 50.5% or 100%, respectively).

Recent studies have reported improved prognosis for recurrent HCC based on the pattern of IHR^5,16^. However, these studies did not clarify the clinical features and pathological course of EHR, with the absence of models to predict EHR after curative treatment for HCC, thus, limiting the early detection of EHR. In our study, we identified the risk factors of EHR after curative hepatectomy for HCC and used these factors to stratify the risk for EHR into four levels. Our findings highlighted the necessity for the development of a predictive score based on risk stratification to inform optimal surveillance for prompt detection of EHR to improve patient outcomes.

The current results of HCC recurrence and EHR development rates suggested some distinction from our previous study of HCC patients who underwent RFA^17^. In that study, the median times to first HCC recurrence and EHR after RFA were 1.75 and 2.68 years, respectively. Moreover, the 1-, 3-, 5-, 8-, and 10-year rates of EHR development were 1.0%, 2.9%, 8.1%, 15.7%, and 33.7%, respectively. These rates were comparably lower than those reported after curative hepatectomy.

Regarding the pattern of HCC recurrence, the most common initial site of recurrence was within the intrahepatic area, which was consistent with a previous report^18^. Another study identified IHR as the most common initial site of recurrence, with EHR developing after several treatments for IHR^19^. Uchino et al. reported that 82.2% of patients with HCC with EHR presented with IHR, a finding comparable to those of our previous study^17^. Therefore, multiple IHRs may indicate EHR risk in patients with HCC^20^.

In our study cohort, the lungs were the most common site of EHR (42.6%). Thoracic metastases, which included the lungs and the vertebrae of the thoracic spine, were, thus, relatively common as previously reported^21^. Thoracic metastases reflect a systemic involvement with poor prognosis, as they are largely not curable. Of further concern is the fact that thoracic metastases may not be detected using conventional surveillance methods that rely on abdominal imaging. Therefore, the use of chest CT images should be included in the surveillance strategy for patients at risk for EHR after curative hepatectomy for HCC for the early detection of thoracic metastases. In addition, the rate of EHR at the time of the initial recurrence of HCC after hepatectomy was 34%, with 38.3% of these patients having no sign of IHR at the time of EHR diagnosis. Therefore, even if intrahepatic HCC lesions are stable, close surveillance for possible development of EHR may be necessary.

Regarding the risk factors for EHR, macrovascular invasion, pathologic mUICC stage (III, IVa), large tumor size (sum > 7 cm), surgical margin involvement, and necrotic HCC were associated with EHR after curative hepatectomy. Vascular invasion is a well-known prognostic indicator of HCC. Natsuizaka et al. showed that vascular invasion was more frequently observed in patients with EHR at the first diagnosis of HCC^1^. Senthilnathan et al. also reported a two-fold increase in EHR in the presence compared to the absence of portal vein invasion (24% *versus* 12%)^22^. Yang et al. reported that EHR was more common among patients with vascular invasion, intrahepatic metastasis, and more advanced HCC stages^11^. A recent study revealed that the presence of tumor necrosis was associated with an advanced tumor stage, HCC recurrence, and patient survival after curative hepatectomy for HCC^23^. In agreement with these findings, we also identified that necrotic HCC was associated with a high rate of EHR. HCC tumors > 6 cm in size were also predictive of EHR after curative resection for HCC, exhibiting similar results to those of a previous study^24^. We identified involvement of the margin of resection as a significant risk factor of EHR (HR: 4.035, 95% CI: 1.28–12.725), which was consistent with the findings of a previous study^25^.

In addition to the aforementioned risk factors for EHR, the first recurrence free survival of < 12 months (HR: 5.748, 95% CI 3.787–8.722, *p* < 0.001) and the serum AFP level > 400 IU/mL at the time of first recurrence during follow-up after curative hepatectomy (HR: 3.127, 95% CI 1.935–5.057, *p* < 0.001) were significantly associated with EHR according to the Kaplan–Meier analysis results. These findings were consistent with those reported by Kim et al. who reported that EHR developed more frequently in patients with early HCC recurrence^26^. They suggested that aggressive tumor pathology was, therefore, a risk factor of early HCC recurrence. Recent studies have shown that high AFP levels were independent risk factors of HCC invasiveness^27–30^. Similarly, our previous study on EHR in RFA for HCC also demonstrated an association between the AFP level and HCC recurrence when the AFP level was > 400 IU/mL, in line with our findings^17^.

After performing multivariate analysis, we found that the presence of EHR was significantly associated with the overall survival rates. Furthermore, pathologic mUICC stage (III, IVa) and surgical margin involvement, which were risk factors of EHR, were also related to the overall survival rate. This finding suggested a close correlation of the EHR with the overall survival rates. Moreover, HCC beyond Milan criteria was associated with the risk of developing HER, as previously reported^31,32^.

Regarding the early EHR, apart from the mentioned risk factors (i.e., advanced pathologic mUICC stage, surgical margin involvement, and macrovascular invasion), the serum ALP levels and absence of fatty change were also significantly associated with early EHR. Although there is still controversy concerning the association between the ALP level and HCC prognosis, a recent study showed that the serum ALP levels increase in liver disease and may reflect liver injury^33^. A recent meta-analysis showed that high pre-treatment ALP levels were significantly associated with poor overall survival rate (HR: 1.14, 95% CI: 1.10–1.18) and RFS (HR: 1.78, 95% CI: 1.37–2.31)^34^. This finding was concordant with our present result presenting an association of higher ALP level with early EHR. The frequency of diffuse steatosis in early HCCs peaks at a diameter of approximately 1.5 cm, decreasing as a function of increasing tumor size and grade^35^. Thus, diffuse fatty change is uncommon in HCCs > 3 cm and with progressing HCC disease status, and is also not usually observed in patients with poorly differentiated HCC^35,36^. Therefore, the absence of fatty change in the liver with HCC is associated with the tumor aggressiveness. In our study, the proportion of patients with the absence of fatty change in the liver was higher for the early EHR than for the non-early EHR group.

We stratified the risk for EHR into four levels based on the number of predictive factors present for EHR. We demonstrated that the cumulative rates of EHR and the median time to her were correlated with these four risk levels. Moreover, we showed that the overall survival rates were correlated with risk levels representing dismal prognosis in patients with EHR. Further, to assess the calibration of our model, the Brier score was calculated (Supplementary Table S3) and showed fine correspondence. Our novel parametric model, albeit simple, could assist clinicians in identifying patients at high risk for EHR before surgery.

We finally noted that 54.8% of the participants in the early EHR group exhibited EHR at the time of first recurrence. This underlines the importance of identifying patients who are at high risk for early EHR, which may be informative concerning the best strategy for surveillance after surgery for the rapid identification of EHR development.

The limitations of our study should be noted in the interpretation of results. First, the diagnosis of EHR was mostly based on medical imaging and, thus, the possibility of other primary cancers from an origin other than the liver could not be completely ruled out. However, in circumstances when the origin of the tumor was not certain, pathologic confirmation was performed at the discretion of the treating physician. Second, this was a retrospective study based on medical records from patients enrolled from two tertiary hospitals. Therefore, the effect of bias on results cannot be denied. Moreover, as this was a retrospective study, there was no concrete treatment algorithm strategy and criteria for resection. Third, there was no uniform post-treatment or surveillance schedule, and the surveillance modality used for each patient was at the discretion of the treating physician. Moreover, patients underwent different treatment modalities for local HCC recurrence depending on the tumor and patient status. There may be diverse conditions concerning the tumor stage, liver reserve function, and patients’ physical performance status. However, we tried to overcome these limitations by using a considerable number of patients with a long-term follow-up duration in multiple tertiary centers. Finally, it might have been insufficient to predict extrahepatic recurrence of HCC using only our model in all patients with HCC. There is a need to conduct a well-organized prospective study to validate more effective methods of EHR prediction and management.

In conclusion, we present a simple parametric model to predict EHR after curative hepatectomy for HCC. This tailored approach may be useful for the early detection of EHR and permit a more refined estimation of risk on an individual basis.

## Methods

### Patients

Between January 2004 and December 2013, 493 consecutive patients who underwent surgical resection for HCC at two tertiary hospitals were evaluated for study enrolment (Supplementary table S4). The inclusion criteria were as follows: age ≥ 18 years; and HCC diagnosis. Surgical resection of HCC was determined by physician’s decision regarding tumor stage and patient’s physical status. There had been no changes in the keynote of surgical resection during study period in both centers. Patients with hepatic tumors other than HCC and those with follow-up duration shorter than 90 days were excluded. After screening, 398 patients were enrolled (Supplementary Fig. S1). Baseline clinical and tumor characteristics, resection method, pathological findings, status of recurrence, and RFS were assessed retrospectively.

### Statement of ethics

The design of our cohort study was approved by the Institutional Review Board of Chonnam National University Hospital (IRB No. CNUH-2019-203). Owing to the retrospective design of our study and the use of de-identified data, the requirement for informed consent was waived under approval of the Institutional Review Board of Chonnam National University Hospital. The study was performed in compliance with the tenets of the Helsinki Declaration of 1975.

### Data collection

The following information was extracted from patients’ medical records for analysis: age, sex, underlying diseases (including hepatitis infection status and alcohol use), blood chemistry [including the Child–Pugh and model for end stage liver disease scores], serum AFP level, pathological findings (tumor size, histological grade, micro-vascular invasion, and presence of satellite lesions), and abdominal imaging for tumor staging at the time of HCC diagnosis. Data on blood chemistry, serum tumor markers, and abdominal imaging obtained at each follow-up visit were also obtained for analysis. Measured data at the time of first recurrence were also evaluated. Survival was defined as the time interval between the date of HCC diagnosis and that of death or last follow-up examination.

### Baseline HCC staging, curative hepatectomy, and follow-up

HCC was diagnosed according to the guidelines of the Korean Liver Cancer Study Group and the National Cancer Center^37^. HCC staging at the time of diagnosis was determined using the mUICC staging system^38^ and the Barcelona Clinic Liver Cancer (BCLC) classification system^39^. Milan criteria were based on radiologic findings at initial diagnosis of HCC^40^. Abdominal CT or magnetic resonance imaging (MRI) and assessment of serological tumor markers were routinely performed at 1 month after surgical resection and at each 3–6-month follow-up visit.

The tumor size was measured by radiologic modalities with CT or MRI. The histological differentiation of HCC was graded according to the criteria of Edmondson and Steiner^41^. The presence of macrovascular invasion was detected on CT imaging or MRI, while microvascular invasion was defined as invasion of vascular structures on microscopic analysis of resected tumor specimens. Bile duct invasion and tumor necrosis were confirmed from pathological findings of resected tumor specimens.

Post-operative complications after surgical resection were analyzed (Supplementary Table S5) and treatment strategies after first intra-and/or extra-hepatic recurrence and extrahepatic recurrence were also assessed (Supplementary Fig. S2). Overall survival days were defined as the time interval between the date diagnosis of HCC and the date of death or last follow-up examination. The cause of death of each patient is presented in Supplementary Table S6.

### Diagnosis of EHR

Most cases of EHR were diagnosed during routine follow-up studies, with few cases diagnosed during evaluation of new onset symptoms or based on significant elevation in AFP or serial AFP without definite intra-hepatic lesions. The diagnosis of EHR was confirmed by contrast-enhanced CT imaging or MRI, and by pathological examination in some patients. In addition, bone scintigraphy, positron emission tomography-CT, and brain MR or CT imaging were also performed at the discretion of the treating physician. Chest radiographs were obtained routinely at the time of admission and when pulmonary symptoms were present. Early EHR was defined as EHR developing within the 1st year after initial curative hepatectomy.

### Statistical analysis

The data are presented as means ± standard deviations or as medians and ranges, as appropriate for the data type and distribution. Univariate analyses were performed using chi-squared test or student’s *t*-test, as appropriate. Variables with a *p*-value < 0.05 on univariate analyses were included in a multivariate logistic regression analysis to identify factors predictive of EHR. The multivariable Cox regression model was built using stepwise backward selection of variables, with variables having a *p-*value < 0.05 retained as predictive factors. The risk levels of EHR were defined based on the number of risk factors present, with Kaplan–Meier survival curves constructed for each risk level. An equivalent of the concordance statistic for use with Cox regression was calculated by Harrell’s C-index.

All statistical analyses were performed using SPSS software (version 25.0, IBM Corp., Armonk, NY, USA).

## Supplementary Information

Supplementary Information.

## Data Availability

The datasets generated during and/or analyzed during the current study are available from the corresponding author on reasonable request.
