# Supplementary material for: Simple parameters predicting extrahepatic recurrence after curative hepatectomy for hepatocellular carcinoma
Source: Sci Rep. 2021 Jun 21;11:12984. doi: 10.1038/s41598-021-92503-6 (PMC8217564; doi:10.1038/s41598-021-92503-6)
Supplement: Supplementary file 1 — Supplementary Information. [file 41598_2021_92503_MOESM1_ESM.docx]

**Simple parameters predicting extrahepatic recurrence after curative hepatectomy for hepatocellular carcinoma**

Running head: Parameters of extrahepatic recurrence

Jae Hyun Yoon, M.D.^1^, Won Jae Lee, M.D.^1^, Sun Min Kim, M.D.^1^, Kwang Tack Kim, M.D.^1^, Sung Bum Cho, M.D., Ph.D.^2^, Hee Joon Kim, M.D.^3^, Yang Seok Ko, M.D., Ph.D.^4^, Hyun Yi Kook,^5^ Chung Hwan Jun, M.D.^6*^, Sung Kyu Choi, M.D., Ph.D.^1*^, Ban Seok Kim, M.D.^1^, Seo Yeon Cho, M.D.^1^, Hye-Su You, M.D.^1^, Yohan Lee, M.D.^1^, Seyeong Son, M.D.^1^

^1^Department of Gastroenterology, Chonnam National University Hospital and Medical School, Gwangju, South Korea

^2^Department of Gastroenterology, Hwasun Chonnam National University Hospital and Medical School, Hwasun, South Korea

^3^Department of Surgery, Chonnam National University Hospital and Medical School, Gwangju, South Korea

^4^Department of Surgery, Hwasun Chonnam National University Hospital and Medical School, Hwasun, South Korea

^5^Department of Nursing, Chonnam National University, Gwangju, South Korea

^6^Department of Internal Medicine, Mokpo Hankook Hospital, Mokpo, South Korea

***Corresponding authors:**

Chung Hwan Jun, MD

Department of Internal Medicine, Mokpo Hankook Hospital, Mokpo 58643, South Korea

Tel: +82-61-270-5360

E-mail address: [estevanj@naver.com](mailto:estevanj@naver.com)

Sung Kyu Choi, MD, PhD

Department of Gastroenterology, Chonnam National University Hospital and Medical School,

Gwangju 61469, South Korea

E-mail address: choisk@jnu.ac.kr

Tel: +82-62-220-6296

Fax: +82-62-220-8578

**SUPPLEMENTARY MATERIAL**

**Supplementary Fig. S1** Flow chart of patient enrolment in the study.

(Early EHR was defined as development of EHR within 1 year after curative hepatectomy)

HCC, hepatocellular carcinoma; CCA, cholangiocarcinoma; EHR, extrahepatic recurrence.

**
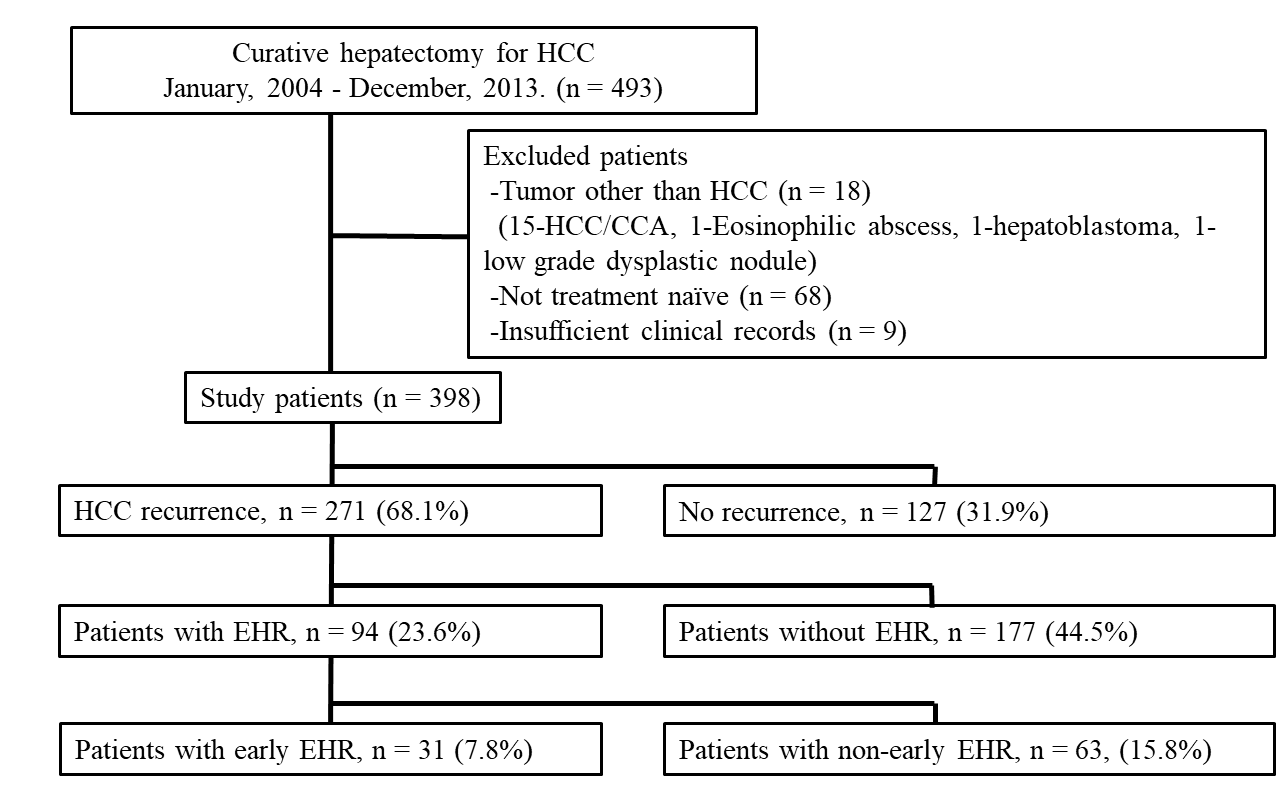
**

**Supplementary Fig. S2** Treatment strategies for (A) first intra- and/or extra-hepatic first recurrence (n=271) and (B) extrahepatic recurrence (n=94) after surgery for HCC

RFA, radiofrequency ablation; TACE, transarterial chemo-embolization; RT, radiotherapy; PEIT, percutaneous ethanol injection therapy; F/U, follow up; CCRT, concurrent chemoradiotherapy.

**(A)**


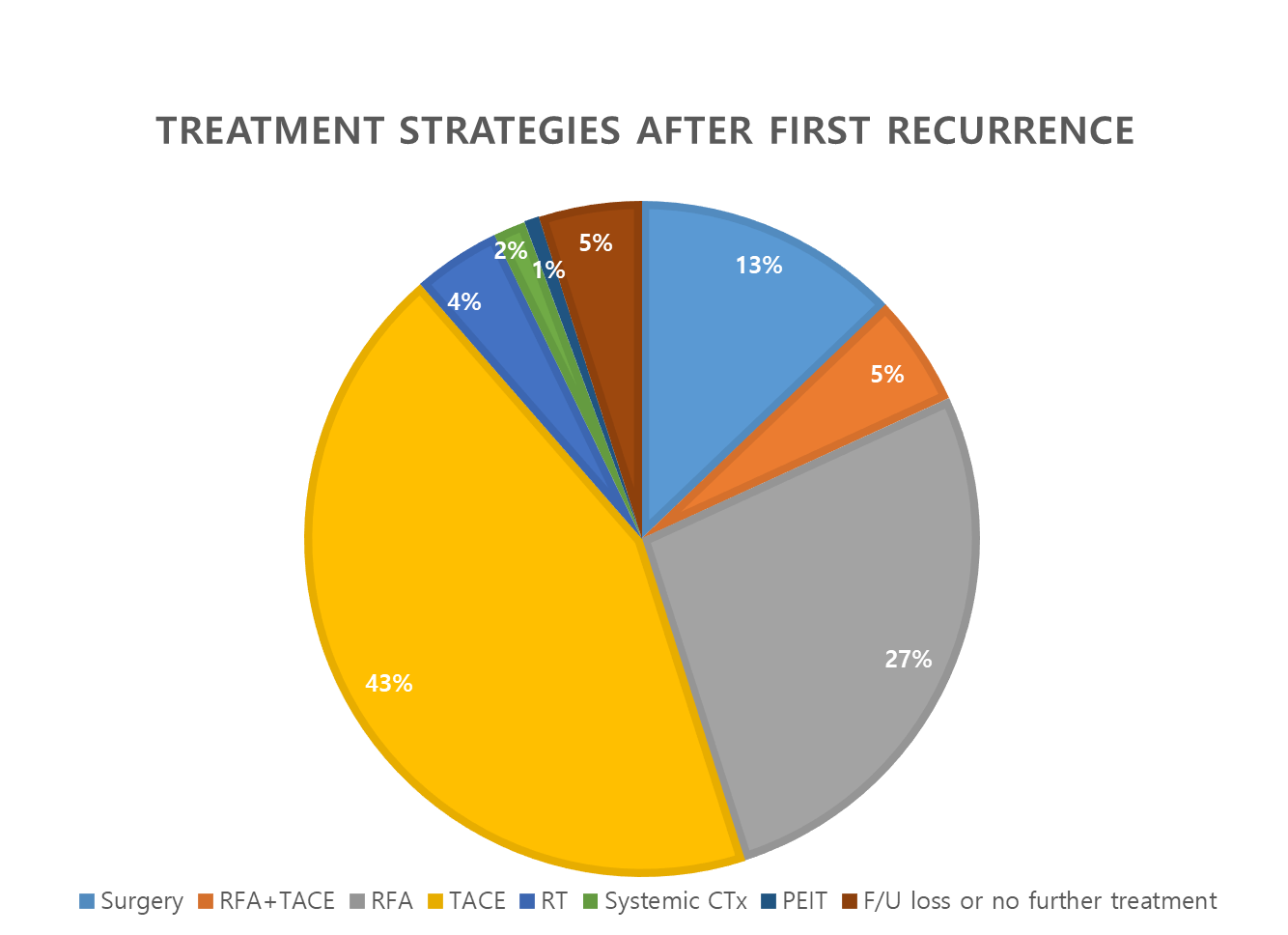


**(B)**

**
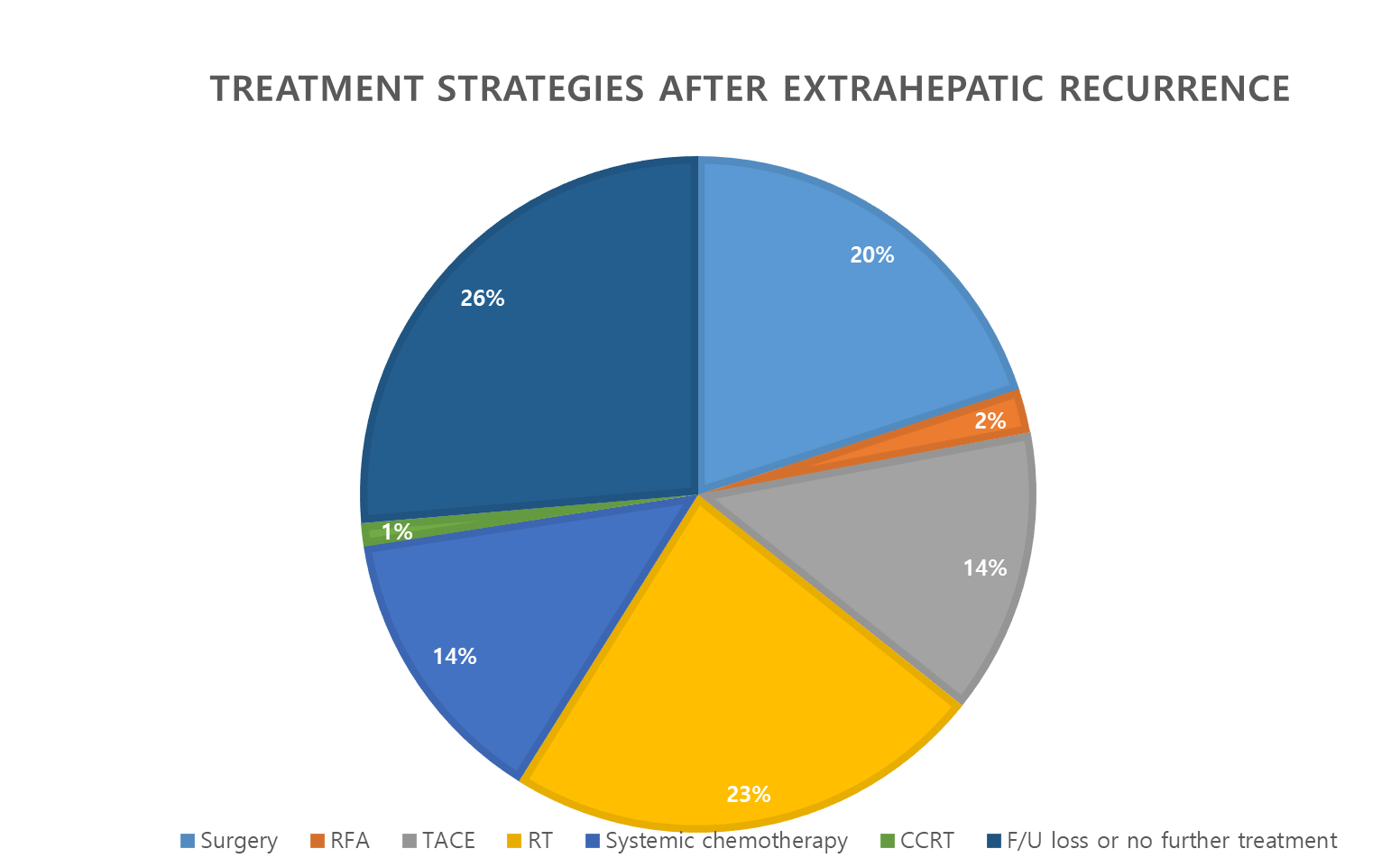
**

**Supplementary Table S1.** **Clinical features of patients with extrahepatic recurrence (n = 94)**

| First recurred site  Single intrahepatic recurrence  Multiple intrahepatic recurrence  Extrahepatic recurrence  Both | 26 (27.7)  36 (38.3)  10 (10.6)  22 (23.4) |
| --- | --- |
| Location of extrahepatic recurrence^1^, n (%)  Lung  Solitary / multiple  Lymph nodes  Peritoneum  Bone  Adrenal gland  Hollow viscus  Spleen  Brain  Heart  Gall bladder | 40 (42.6)  9 (9.6) / 31 (33.0)  18 (19.1)  17 (18.1)  14 (14.9)  9 (9.6)  3 (3.2)  3 (3.2)  2 (2.1)  1 (1.1)  1 (1.1) |
| EHR confined to abdominal cavity / EHR including thoracic cavity / Others | 47 (50.0) / 46 (48.9) / 1 (1.1) |
| Time to extrahepatic recurrence (years, median, range) | 2.06 (0.02-12.19) |
| Intra-hepatic HCC status at diagnosis of EHR (mUICC T stage), n (%)  T0/T1/T2  T3/T4 | 36 (38.3) / 2(2.1) / 8 (8.5)  31 (33.0) / 17 (18.1) |
| EHR as 1^st^ recurrence, n (%) | 32 (34.0) |

^1^ Eleven patients had multiple EHR occurrences (1 patient: multiple lung and bone, 1 patient: multiple lung, bone, and adrenal gland, 1 patient: multiple lung and adrenal gland, 1 patient: multiple lung and peritoneum, 1 patient: multiple lung and brain, 1 patient: multiple lung and gall bladder, 1 patient: multiple lung and heart, 1 patient: bone, lymph nodes, and spleen, 1 patient: lymph node and adrenal gland, 1 patient: lymph node, adrenal gland, and peritoneum, and 1 patient: lymph node and peritoneum)

HCC, hepatocellular carcinoma; mUICC, modified Union for International Cancer Control

**Supplementary Table S2. Comparison of early extrahepatic recurrence and non-early extrahepatic recurrence**

|  | Early extrahepatic recurrence  (n=31) | Non-early extrahepatic recurrence (n=63) | *p*-value |
| --- | --- | --- | --- |
| Tumour number | 1.39 ± 0.80 | 1.29 ±0.79 | 0.101 |
| Tumour size | 6.98 ± 4.59 | 5.15 ± 3.02 | 0.050 |
| Radiologic mUICC stage, n (%)  I / II  III / Iva | 2 (6.5) / 17 (54.8)  8 (25.8) / 4 (12.9) | 8 (12.7) / 42 (66.7)  13(20.6) / 0 (0.0) | **0.022** |
| Pathologic mUICC stage, n (%)  I / II  III / Iva | 1 (3.2) / 10 (32.3)  12 (38.7) / 8 (25.8) | 6 (9.5) / 39 (61.9)  14 (22.2) / 4 (6.3) | **0.005** |
| BCLC stage, n (%)  0 / A  B / C | 1 (3.2) / 22 (71.0)  5 (16.1) / 3 (9.7) | 5 (7.9) / 46 (73.0)  10 (15.9) / 2 (3.2) | 0.496 |
| Edmondson Steiner worst grade, n (%)  1 / 2  3 / 4 | 1 (3.2) / 1 (3.2)  18 (58.1) / 11 (35.5) | 1 (1.6) / 13 (21.0)  37 (59.7) / 11 (17.7) | 0.062 |
| Margin involvement, n (%) | 4 (12.9) | 2 (3.2) | 0.073 |
| Veno-lymphatic involvement, n (%) | 13 (41.9) | 15 (23.8) | 0.071 |
| Serosal invasion, n (%) | 2 (6.5) | 4 (6.5) | 1.000 |
| Bile duct invasion, n (%) | 1 (3.2) | 1 (1.6) | 0.613 |
| Multicentricity, n (%) | 4 (12.9) | 5 (8.1) | 0.457 |
| Satellite nodule, n (%) | 11 (35.5) | 18 (29.0) | 0.527 |
| Presence of fatty change, n (%) | 4 (13.3) | 25 (41.0) | **0.008** |
| Beyond Milan criteria, n (%) | 16 (51.6) | 29 (46.0) | 0.611 |
| Serum AFP at initial diagnosis | 2,583.08 ± 9,332.66 | 1,718.50 ± 7,037.08 | 0.620 |
| Recurrence free survival (median, months) | 8.92 ± 17.14 | 28.11 25.26 | **<0.001** |
| Interval to EHR (median, months) | 5.03 | 54.97 | **<0.001** |
| Serum AFP at 1^st^ recurrence | 4,547.4255 ± 14,460.55 | 494.66 ± 1,766.21 | 0.130 |
| mUICC stage at 1^st^ recurrence, n (%)  I / II  III/ IVa  IVb | 1 (3.2) / 3 (9.7)  7 (9.7) / 3 (9.7)  17 (54.8) | 14 (22.2) / 23 (36.5)  9 (1.4) / 1 (1.6)  16 (25.4) | **0.001** |
| First recurred site  Single intrahepatic recurrence  Multiple intrahepatic recurrence  Extrahepatic recurrence  Both | 4 (12.9)  10 (32.3)  3 (9.7)  14 (45.2) | 22 (34.9)  26 (41.3)  7 (11.1)  8 (12.7) | **0.004** |
| Portal vein thrombosis at 1^st^ recurrence, n (%) | 1 (3.2) | 4 (6.5) | 0.516 |

mUICC, modified Union for International Cancer Control; BCLC, Barcelona Clinic Liver Cancer; AFP, alpha-fetoprotein

**Supplementary Table S3.** **Calibration of the model with comparison between observed and predicted curves.**

|  | Brier score  (mean value) | Standard error | 95% CI |
| --- | --- | --- | --- |
| 1 year | 0.061 | 0.010 | 0.042-0.080 |
| 2 year | 0.087 | 0.011 | 0.066-0.108 |
| 3 year | 0.103 | 0.011 | 0.080-0.125 |
| 4 year | 0.112 | 0.012 | 0.089-0.135 |
| 5 year | 0.129 | 0.013 | 0.104-0.154 |
| 6 year | 0.149 | 0.014 | 0.123-0.176 |
| 7 year | 0.180 | 0.017 | 0.147-0.213 |
| 8 year | 0.188 | 0.019 | 0.150-0.226 |
| 9 year | 0.180 | 0.018 | 0.144-0.216 |
| 10 year | 0.208 | 0.027 | 0.155-0.262 |

**Supplementary Table S4. Comparison of patients group characteristics between two centers.**

|  | | Chonnam national university hospital (n=202) | Hwasun Chonnam national university hospital (n=196) | *p*-value |
| --- | --- | --- | --- | --- |
| Age (years) | 59.15 ± 9.88 | | 57.58 ± 10.48 | 0.124 |
| Male (n, %) | 172 (85.1) | | 172 (87.8%) | 0.448 |
| ALP (U/L) | 90.16 ± 36.52 | | 89.44 ± 33.12 | 0.838 |
| Albumin (mg/dL) | 4.25 | | 4.34 | **0.048** |
| ALBI grade ≥ 2, n (%) | 42 (21.0%) | | 28 (14.3%) | 0.080 |
| Serum AFP (IU/mL) | 871.73 ± 3158.73 | | 2005.01±7825.29 | 0.061 |
| PIVKA-II (mAU/mL) | 926.08 ± 1873.00 | | 1339.95 ± 3314.44 | 0.619 |
| Tumor size | 4.431 ± 2.69 | | 4.68 ± 2.99 | 0.391 |
| Tumor numbers | 1.30 ± 1.00 | | 1.24 ± 0.58 | 0.440 |
| BCLC stage, n (%)  0 / A / ≥B | 19 (9.5) / 158 (78.6) / 24 (11.9) | | 21 (10.7) / 144 (73.5) / 31 (15.8) | 0.230 |
| Pathological mUICC stage, n (%)  I / II / ≥III | 27 (13.6) / 111 (56.1) / 60 (30.6) | | 23 (11.9) / 121 (62.7) / 49 (25.4) | 0.248 |
| Radiological mUICC stage, n (%)  I / II / ≥III | 30 (14.9) / 138 (68.7) / 33(16.4) | | 28 (14.3) / 126 (64.3) / 41 (20.9) | 0.354 |
| Beyond Milan criteria, n (%) | 56 (27.9%) | | 64 (32.7%) | 0.299 |
| Metastatic lymph nodes, n (%) | 1 (0.5) | | 1 (0.5) | 0.986 |
| Macrovascular invasion, n (%) | 5 (2.5) | | 9 (4.6) | 0.252 |
| Hospital stay, days (median, range) | 13 (4-63) | | 13 (4-69) |  |
| Time to first recurrence, months  (median, range) | 25.57 (0.16-157.91) | | 30.84 (0.23-142.95) | 0.364 |
| Time to extrahepatic recurrence, months (median, range) | 60.08 (2.17–177.40) | | 67.22 (0.23-177.40) | 0.182 |
| Extrahepatic recurrence | 53 (26.2) | | 41 (20.9) | 0.212 |
| Follow-up duration, months  (median, range) | 65.23 (2-177) | | 70.98 (2-177) | 0.224 |

**Supplementary Table S5.** **Post-operative complication after surgical resection of hepatocellular carcinoma (n=398).**

| Liver function deterioration  Ascites requiring drainage  Varix bleeding  Hepatic hydrothorax  Hepatic encephalopathy  Pneumothorax  Pneumonia  Wound infection | 13 (3.27%)  1 (0.25%)  12 (3.02%)  3 (0.75%)  1 (0.25%)  4 (1.01%)  4 (1.01%) |
| --- | --- |
| Ileus  Biloma requiring drainage  Bile peritonitis  Gastric ulcer  Operation site bleeding | 4 (1.01%)  5 (1.26%)  1 (0.25%)  1 (0.25%)  3 (0.75%) |

**Supplementary Table S6.** **Comparison of the cause of death between patients group categorized by survival duration of 1 year (n=198).**

|  | Survival duration  ≤ 1year (n=22) | Survival duration  > 1year (n=176) |
| --- | --- | --- |
| Hepatocellular carcinoma | 21 (95.5%) | 156 (88.6%) |
| Malignancy other than hepatocellular carcinoma | 1 (4.5%) | 9 (5.1%) |
| Cerebrovascular event | - | 4 (2.3%) |
| Trauma | - | 4 (2.3%) |
| Infection | - | 3 (1.7%)s |
